# Pathogen Adaptation to American (*Rpv3-1*) and Eurasian (*Rpv29*) Grapevine *Loci* Conferring Resistance to Downy Mildew

**DOI:** 10.3390/plants11192619

**Published:** 2022-10-05

**Authors:** Elena Marone Fassolo, Beatrice Lecchi, Demetrio Marcianò, Giuliana Maddalena, Silvia Laura Toffolatti

**Affiliations:** Department of Agricultural and Environmental Sciences, University of Milan, 20133 Milano, Italy

**Keywords:** plant–pathogen interaction, oomycete, disease resistance, durability of plant resistance, disease intensity

## Abstract

Durable resistance is a key objective in genetic improvement for disease resistance in grapevines, which must survive for years in the field in the presence of adaptable pathogen populations. In this study, the adaptation of 72 Northern Italian isolates of *Plasmopara viticola*, the downy mildew agent, has been investigated into Bianca, possessing *Rpv3-1*, the most frequently exploited resistance *locus* for genetic improvement, and Mgaloblishvili, a *Vitis vinifera* variety possessing the newly discovered *Rpv29 locus*. Infection parameters (latency period, infection frequency, and disease severity) and oospore production and viability were evaluated and compared to those of Pinot noir, the susceptible reference. The expected levels of disease control were achieved by both resistant cultivars (>90% on Bianca; >25% on Mgaloblishvili), despite the high frequency of isolates able to grow on one (28%) or both (46%) accessions. The disease incidence and severity were limited by both resistant cultivars and the strains able to grow on resistant accessions showed signatures of fitness penalties (reduced virulence, infection frequency, and oospore density). Together, these results indicate an adequate pathogen control but suitable practices must be adopted in the field to prevent the diffusion of the partially adapted *P. viticola* strains to protect resistance genes from erosion.

## 1. Introduction

The Eurasian grapevine *Vitis vinifera* L. [1] is affected by numerous pests and diseases that greatly reduce production each year. The main diseases affecting grapevine, in the current climatic conditions and scenario projections for 2050, are downy and powdery mildews and gray mold, all caused by fungi [2]. Together, these three diseases are responsible for the application of about 20 fungicide treatments in a relatively short period (April–September), from grape sprouting to harvest. Downy mildew caused by the oomycete *Plasmopara viticola* (Berk. et Curt.) Berl. and De Toni is the most important grapevine disease in intermediate to hot climates with sub-humid to humid conditions, such as Northern Italian ones [3,4]. *P. viticola* is an obligate parasite that infects receptive grapevine tissues through stomata by forming an intercellular mycelium with haustoria for nutrition [5]. The pathogen asexually reproduces by forming sporangiophores with sporangia, which in turn produce zoospores for new infection cycles, starting from 4–5 days of incubation at optimal temperatures (22–25 °C) and high relative humidity (>60%) [6,7].

Cultural practices alone are hardly effective to control grapevine downy mildew, and disease control mostly relies on the use of fungicides. However, the progressive reduction in the fungicide modes of action and the widespread presence of fungicide resistance in the pathogen populations are challenging disease management [8].

The cultivation of resistant vines, with qualitative characteristics suitable for winemaking and direct and indirect consumption, would entail numerous advantages in eco-toxicological and economic terms. Traditionally, breeding programs for resistance to downy mildew are based on the crossing of *V. vinifera* varieties with grapevine species that belong to areas where the host plants have co-evolved with *P. viticola* or a similar species [9]. The intense breeding activity carried out for over a century allowed breeders to obtain numerous varieties that couple the quality traits of *V. vinifera* and the resistant traits of American or Asian species [10]. At least 28 QTLs (quantitative trait loci) associated with *P. viticola* resistance are known in several *Vitis* species and hybrids (https://www.vivc.de/loci, accessed on 9 May 2022). Of these, the *Rpv3* locus, found in North American wild species (*V. labrusca*, *V. lincecumii*, *V. riparia*, and *V. rupestris*) [11], is the most widespread, since it can be found in most of the resistant cultivars registered on the Vitis International Variety Catalogue VIVC [12]. *Rpv3* resistance is linked to the occurrence of a hypersensitive response (HR) triggered by the products of two nearby TIR-NB-LRR genes and mediated by the synthesis of reactive oxygen species [13,14,15]. Despite this very effective mechanism of resistance, very limited sporulation can sometimes be seen in this case. More recently, three new genomic loci (*Rpv29*, *Rpv30*, and *Rpv31*) associated with a different mechanism of resistance, have been found in the *V. vinifera* germplasm of Georgia (South Caucasus). These *loci* induce partial resistance, where the pathogen growth and sporulation are significantly reduced compared to that in susceptible grapevine varieties [16,17].

The durability of resistance is a key factor for disease management, especially for tree crops, which must be productive for years and must be preserved by adopting suitable strategies, such as pyramiding different resistance genes in a single cultivar [18]. A breakdown of resistance to *Rpv3* has been already found in *P. viticola* strains from the Czech Republic, Italy, and Germany [14,19,20,21,22]. In these reports, the resistant accession showed the same disease intensity as the susceptible reference. The characterization of pathogen populations is very important to deploy the different QTLs for plant genetic improvement [23]. *P. viticola* produces both asexual (zoospores differentiated in sporangia) and sexual (oospores) spores for infection and survival, respectively, and possesses a polycyclic behavior, causing primary and secondary infection cycles within the same grapevine vegetative season. This favors the selection and diffusion of adapted pathogen strains.

The aim of this study was to gain an insight into the potential durability of resistance to two separate QTLs, *Rpv3-1* and *Rpv29*, through the evaluation of adaptation of *P. viticola* strains in northern Italy. *P. viticola* strains (72) were isolated from different regions and characterized for several fitness parameters such as infectivity (latency period and disease incidence and severity), estimated from the presence of asexual reproductive structures (sporangiophores bearing sporangia), and sexual reproduction (oospores) on three cultivars with different genetic backgrounds and susceptibility profiles: Pinot Noir (*V. vinifera*, susceptible), Bianca (resistant interspecific hybrid harboring *Rpv3*-1 QTL), and Mgaloblishvili (*V. vinifera*, resistant variety with *Rpv29* QTL) [13,15,24]. The pathogenicity of each *P. viticola* strain on the three different grapevine varieties was determined according to the leaf surface covered by sporulation at 5–13 days after inoculation. Furthermore, the production and viability of oospores (sexual spores produced by the pathogen for overwintering) by strains able to grow on resistant cultivars were assessed to evaluate whether survival of the pathogen is hampered in resistant varieties.

## 2. Results

### 2.1. Infection Parameters of the Isolates

The average IF (infection frequency, expressed as a percentage) and PSA (percentage of sporulating area) values ± standard deviation (SD) recorded by the isolates on PN (*V. vinifera* cv Pinot noir, susceptible to *P. viticola*), MG (*V. vinifera* cv Mgaloblishvili, resistant to *P. viticola*), and B (*V. vinifera* interspecific hybrid cv Bianca, resistant to *P. viticola*) are reported in Table 1. The latency period (LP) length, measured in degree-days (DD), was statistically analogous (*p* = 0.24) among cultivars (128 ± 31 DD on PN; 131 ± 32 DD on MG; 142 ± 45 DD on B).

**Figure 1:**
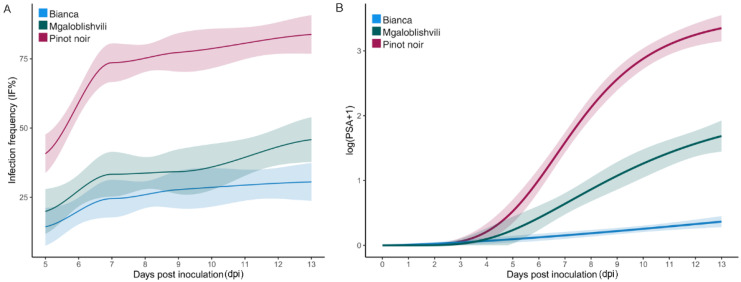
Fitted curves of IF (**A**) and log(PSA + 1) (**B**) values recorded on Bianca, Mgaloblishvili, and Pinot noir from 5 to 13 dpi by *P. viticola* isolates. IF lines were obtained through local polynomial regression fitting using loess() function in R, while PSA curves were obtained according to log-logistic regression model predictions. Light-colored ribbons show 95% confidence intervals for fitted curves.

The IF values recorded by *P. viticola* isolates for each dpi on PN (41% < IF < 84%) were significantly higher (*p* < 0.0001) than those observed on MG (20% < IF < 46%) and B (14 < IF < 31%), that in turn showed statistically analogous values (*p* > 0.06) (Table 1; Figure 1A).

The average PSA range between 5 and 13 dpi was 1–39% on PN, 0.1–0.7% on B, and 0.4–13% on MG. The log-logistic model described satisfactorily the transformed PSA values (log(PSA + 1)) over dpi (lack-of-fit test: F-value = 0.17; df = 3; *p* = 0.92) and allowed calculation of the regression parameters reported in Table 2. Except for *d* (upper asymptote) and *e* (t_50_) parameters estimated for B (t-statistics *p* > 0.05), the remaining parameters adequately described the time–response curves for MG and PN (Figure 1B). Multiple comparisons among the parameters of the three curves (Table 2) showed that PN and B significantly differed for the slope of the curve (*b*) that was higher on PN than on B (Figure 1B), with MG showing no differences with the other two cultivars. Conversely, PN and MG significantly differed for the upper asymptote (*d*) but not for the slope. Indeed, at 13 dpi the average PSA values were 39% on PN, 13% on MG, and 0.7% on B. Statistically analogous t_50_ values were recorded on PN (7 dpi) and MG (9 dpi).

### 2.2. Identification of Potentially Adapted Isolates

**Figure 2:**
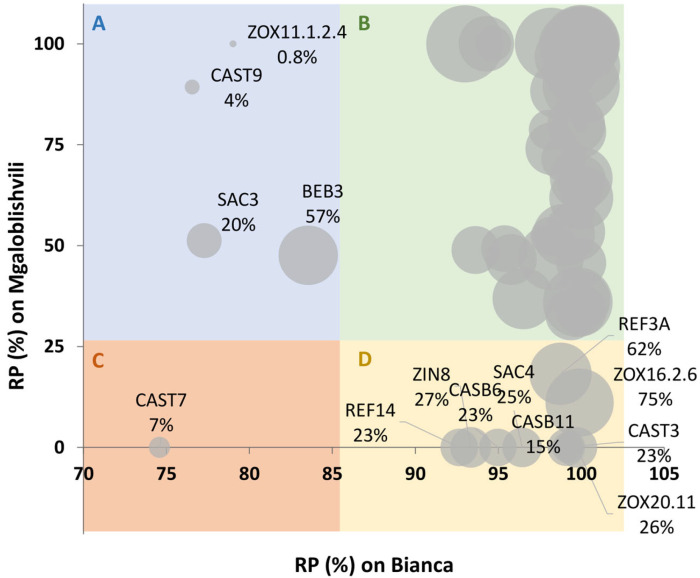
Bubble chart representing the percentage of PSA reduction (RP) on Bianca and Mgaloblishvili compared to Pinot noir. The size of the bubbles represents the PSA value on Pinot noir (the greater the size, the greater the PSA value). Percentages below the isolate codes indicate the PSA values on Pinot noir. Only the codes of adapted *P. viticola* isolates are reported. (**A**) Isolates showing RP < 85% on B and RP > 25% on MG (B-adapted). (**B**) Isolates with RP > 85% on B and RP > 25% on MG (non-adapted). (**C**) Isolate with RP < 85% on B and RP < 25% on MG (B- and MG-adapted). (**D**) Isolates with RP > 85% on B and RP < 25% on MG (MG-adapted).

Looking in detail at the distribution frequencies of the PSA values (Table 3), it can be seen that most of the isolates (63%) grouped in the 25.1–100% range on PN, while only 27 strains (37%) were in the 0.1–25% range. All strains (97%) caused limited sporulation on Bianca (0 < PSA < 5%) apart from two, which fell in the 5.1–10% range. On MG, a wider distribution of the pathogen strains could be observed: 76% of the isolates grouped in the 0–25% PSA interval, 22% grouped in the 25.1–50% interval, and 2% in the 50.1–75% interval. To identify potential adapted (i.e., able to overcome B and MG resistance) strains, the distribution of the RP values (reduction in PSA on B and MG compared to PN, expressed as a percentage) of the isolates was analyzed. The individual PSA values on PN, the RP values on MG and B, and the classification of the isolates as adapted/non-adapted are available in Supplementary Table S1. Only the names of the potentially adapted isolates will be recalled in the text and the figures. The bubble chart distribution of RP values clearly shows the presence of four different groups of strains (Figure 2). Group A includes four strains with RP values on B below 85% that are potentially B-adapted (i.e., able to grow on B). Group B includes 58 non-adapted strains (i.e., strains whose growth is strongly affected by both B and MG). Group C is particularly important since it includes one strain (CAST7) that is adapted to both resistant cultivars (RP < 85% on B and RP < 25% on MG). Group D includes nine strains with RP < 25% on MG that are MG-adapted (i.e., able to grow on MG). It is interesting to notice that the adapted strains showed in most of the cases PSA values as below 27% on PN, the susceptible control (Figure 2; Supplementary Table S1). Only a B-adapted strain (BEB3) and two MG-adapted strains (REF3A and ZOX16.2.6) showed high infectivity (PSA > 57%) on PN (Figure 2).

### 2.3. Infection Parameters of the Isolates Divided into Pathogenicity Classes

Based on pathogenicity tests, the isolates were divided into four pathogenicity classes (PC). In detail, 19 isolates (26%) were able to infect PN alone (PC1), 8 isolates (11%) infected PN and B (PC2), 12 isolates (17%) affected both PN and MG (PC3) and 33 strains (46%) grew on PN, B, and MG (PC4) (Table 4). No significant correlation was found between the geographical origin of the strains (province and vineyard) and PC (*p* > 0.4).

The ranges of LP values were 121–138 DD on PN, 128–131 DD on MG, and 139–154 DD on B. No significant differences were found among LP values of the different PCs (*p* > 0.08).

**Figure 3:**
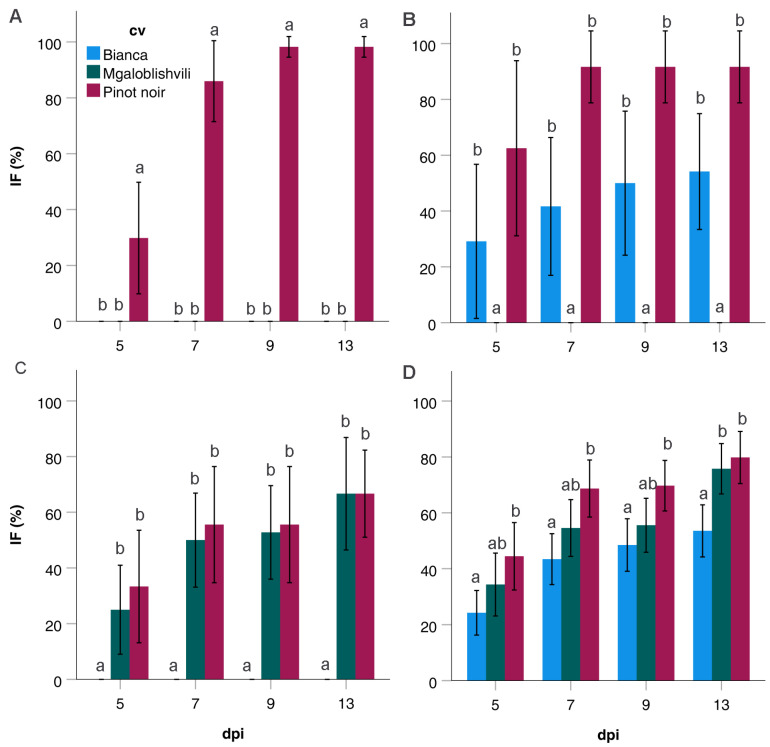
IF values (%) recorded on Bianca, Mgaloblishvili, and Pinot noir from 5 to 13 dpi by *P. viticola* isolates grouped in PC1 (**A**), PC2 (**B**), PC3 (**C**), and PC4 (**D**). Different letters within dpi indicate the existence of significant differences among IF values on the different cultivars (*p* < 0.05).

Considering individual time-points, IF of the strains grouped in the different pathogenicity classes varied among (Figure 3) and within cultivars (Table 1). The IF values of PC2 and PC3 strains were analogous (*p* > 0.05) on PN and B (Figure 3B) or MG (Figure 3C) at all time-points. PC4 strains showed significantly higher IF (*p* < 0.04) on PN than on B at all time-points (Figure 3D). Intermediate IF values were found on MG until 9 dpi (Figure 3D); at 13 dpi, the IF values of MG were significantly higher than those on B. Concerning the individual cultivars, the only significant differences among PC classes were found on PN, where PC1 showed significantly higher IF (*p* < 0.043) than PC3 at 7–13 dpi and PC4 at 9–13 dpi (Table 1).

**Figure 4:**
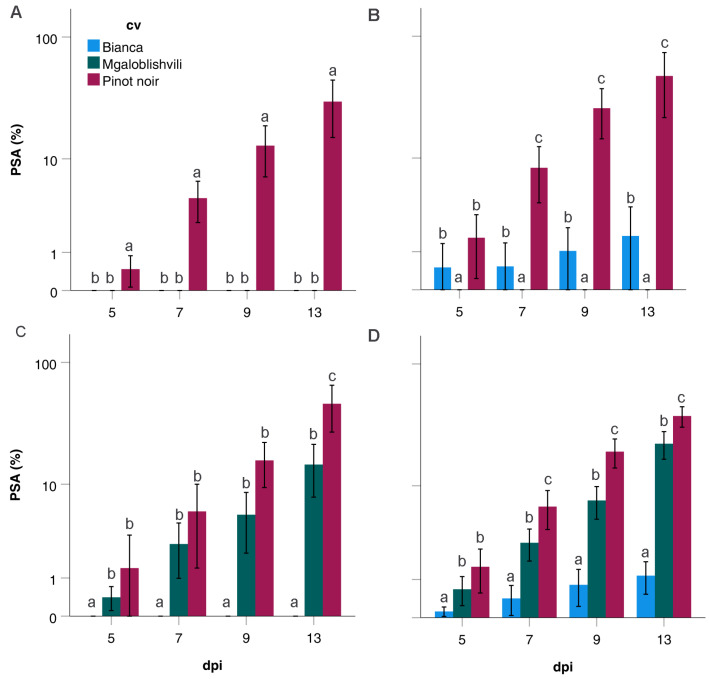
PSA values (%) recorded on Bianca, Mgaloblishvili, and Pinot noir from 5 to 13 dpi by *P. viticola* isolates grouped in PC1 (**A**), PC2 (**B**), PC3 (**C**), and PC4 (**D**). Different letters within dpi indicate the existence of significant differences among IF values on the different cultivars (*p* < 0.05).

The PSA values obtained by the four PCs on the three cultivars are reported in Figure 4. Additionally, in this case, significant differences among cultivars were found in PC4. PC4 strains, in fact, caused significantly lower PSA (*p* < 0.02) on B (at all time-points) and on MG (from 7 to 13 dpi) than on PN (Figure 4D). Statistically analogous PSA values (*p* > 0.07) were found among infecting PCs within cultivars at each dpi (Table 1).

### 2.4. Oospore Production and Viability

The average oospore density (OD) ranged from 6 to 8.2 oospores/cm^2^ on PN, 4.6 to 6.3 oospores/cm^2^ on B, and 4.9 to 5.6 oospores/cm^2^ on MG (Table 5). No significant differences were found among OD values achieved by the different mating types on the individual grapevine cultivars (*p* > 0.4). No significant differences (*p* > 0.42) were moreover found between OD values of CAST1xCAST7 and CASB11xCAST1 mating pairs on the three cultivars. Conversely, CASB11xCAST7 crossing showed significantly lower OD values (*p* = 0.022) on B (OD = 4.6 ± 0.4 oospores/cm^2^) and MG (OD = 5.6 ± 0.6 oospores/cm^2^) than on PN (OD = 8.2 ± 1.9 oospores/cm^2^) (Table 5).

OV (oospore viability, expressed as a percentage) values ranges were 67–87% on PN, 70–87% on MG, and 80–93% on B (Table 5). No significant differences were found either between OV and cultivar (*p* = 0.32) or mating pairs (*p* = 0.59).

## 3. Discussion

The most widespread *V. vinifera* cultivars are commonly susceptible to *P. viticola* and only recently has the partial investigation of the Georgian germplasm allowed the identification of the resistant variety MG and the first QTLs associated with downy mildew resistance (*Rpv29-31*) within the species [16,24]. The unique mechanism of resistance of MG [17,25] makes the cultivar suitable for genetic improvement of grapevine for downy mildew resistance, where stacking different resistance *loci* is fundamental to avoid resistance breakdown (adaptation) by *P. viticola* [10]. Before exploiting resistance *loci*, it is important to characterize the pathogen population for the presence of adapted pathogen strains in the area where the plants will be grown [23]. Here, the ability to infect grapevine accessions with different susceptibility profiles and several fitness components (disease incidence and severity, latency period, t_50_, production, and viability of resting spores) were investigated in *P. viticola* isolates of Northern Italy, an area where downy mildew frequently causes severe damage.

The experimental inoculation of the 72 *P. viticola* isolates on PN (susceptible *V. vinifera* reference), B (interspecific hybrid harboring the *Rpv3.1 locus*), and MG (resistant *V. vinifera* possessing the *Rpv29 locus*) resulted in a reduced infection frequency (IF) and disease severity (PSA) on the two resistant accessions compared to the susceptible one. Nevertheless, while the IF values on B and MG were analogous, the PSA values on MG were higher than those observed on B. IF is the parameter that better unifies the two resistant cultivars and it is also the most indicative parameter for comparing susceptible and resistant accessions; in fact, as a disease incidence index, it quantifies the ability to infect the host as a proportion or percentage of diseased individuals out of the total assessed [26]. By contrast, the disease severity expresses the proportion or percentage of plant tissue affected by the disease. As evaluated in this study, severity measures the extent of the secondary inoculum produced by the pathogen and, indirectly, the extent of colonization.

Concerning the overall disease progress of the pathogen strains, the two resistant accessions did not differ from PN for LP, indicating that when infection occurs, the time to first sporulation is not affected in the selected resistant cultivars, contrarily to what was observed in other studies [27]. What changed between PN and B was the slope of the curve and the disease severity, indicating that the pathogen development was both slowed down and limited. However, the slope of the curve and t_50_ (the time to reach 50% of the total PSA) of MG were similar to those of PN, even if the final disease severity was three times reduced. This indicates that the response of MG is based on a reduction rather than a slowdown of the pathogen infection. Indeed, the time-course investigation of *P. viticola* development in leaf tissues previously showed that the development of the pathogen is identical in PN and MG until 3 dpi, and it was only from this time-point onwards that the vegetative growth of the pathogen was hampered [17]. The lower PSA and slope of the disease progress curve observed in B indicate a stronger limitation of the rate of disease development after the initial infection. This can be explained by the different characteristics of the two resistant varieties: the mechanism of resistance of B is based on the hypersensitive response (HR) and the presence of very low sporulation in the infected areas [12,15,17]; MG response to *P. viticola*, instead, does not lead to HR but to a limitation of the pathogen growth starting from 3 dpi [17].

RP can be proposed as a new parameter for the evaluation of *P. viticola* adaptation to plant resistance in addition to disease severity, which is commonly used as a phenotyping tool for the identification of resistant traits in grapevine [28]. In fact, it easily estimates the measure in which the growth of the strain is affected by the plant considering, at the same time, the virulence level of the strain evaluated on a susceptible reference. Indeed, as visible in the PSA values achieved on PN, the different strains can be characterized by different infection capabilities (virulence). Looking at the disease severity (PSA) values of a strain on a resistant accession without considering its virulence on a susceptible reference can lead to a misidentification of an adapted strain. RP, on the contrary, enables the easy identification of potentially adapted strains. For instance, if a strain possesses similar PSA values on both susceptible and resistant plants (low RP value) it can be a candidate-adapted strain. On the contrary, if a strain possesses a high PSA on the susceptible plant and a low PSA on a resistant plant (high RP value), then it is likely a non-adapted strain. Furthermore, a simple bubble-chart representation of these values such as that performed in this study, easily shows whether the strain possesses a low or high virulence.

It could be argued that MG is not suitable for the genetic improvement of grapevine for downy mildew resistance since the disease severity is higher than that on B. However, it must be considered that B is an interspecific hybrid harboring the resistance genes of the American vines that co-evolved with *P. viticola*, while MG is a pure *V. vinifera* variety that met the pathogen only after its introduction into Europe at the end of the XIX century [29]. The reason for the presence of resistance traits in MG has not been elucidated yet. What is known is that during the interaction with *P. viticola,* MG overexpresses genes encoding for DAMP, PAMP and an effector receptor and other genes associated with signal transduction and synthesis of chemical (terpenes) and physical (cell wall thickening) barriers [17,25,30]. The possibility of exploiting MG genes for resistance to *P. viticola* has two advantages: the first is that crossings are easily made within the species (and not between species, as happens with other *Vitis* species); the second is that MG can be used for stacking new traits into already obtained crosses, contributing to the achievement of a durable resistance [31].

Looking in detail at the behavior of the single isolates on the resistant cultivars, it was possible to see that about 40% of the strains show no signatures of adaptation, since they were not able to infect B nor MG. Of the remaining strains, only 12.5% showed adaptation to MG and 5.6% to B, as demonstrated by the RP values below 25% for MG and 85% for B. Only one strain (CAST7) showed adaptation to both B and MG and should be better characterized because it groups in PC4. Almost all the adapted strains were not very aggressive, as testified by the reduced growth on the reference cultivar PN, and therefore they could possess fitness penalties compared to non-adapted strains within the pathogen population in the field. The studies on the effector array of *P. viticola* during the interaction with *vinifera* and non-*vinifera* species are under constant evolution [25,32,33,34]. It would be interesting in the future to identify the effectors expressed by these two strains.

Pathogenicity tests showed that most of the isolates (74%) were able to infect two (PC2 and PC3 strains) or three (PC4 strains) cultivars, while only 26% of the isolates (PC1) were able to infect PN alone. Even if PC3-4 strains were able to infect the two resistant cultivars, it must be pointed out that they induced lower disease intensities. Indeed, PC2-4 showed a reduced PSA on B and MG compared to PN, and PC4 was also characterized by a reduced IF on B. Considering the results obtained at each time-point on the individual cultivars, no significant differences could be found among the different pathogenicity classes; for instance, PC1-4 showed analogous LP length, IF, and PSA values. Since the different PCs had analogous disease parameters on the individual cultivars, we can assume that PCs have similar fitness and that the lower PSA on B and MG compared to PN is a consequence of the resistant response of the plants. The only significant difference among PCs was found in the IF values on PN, where PC3 individuals showed a 20–33% significantly lower disease incidence than PC1, with PC2 and PC4 in between. This could indicate that the strains able to infect plants with *Rpv29* possess a slight fitness penalty that leads to a limited number of infections on PN.

The oospore density achieved by experimentally inoculating *P. viticola* mating pairs on detached grapevine leaves in this study (5–8 oospores/cm^2^) was quite a lot lower than that obtained from naturally infected leaves in vineyards in a previous study (308.5 oospores/cm^2^) [35]. This difference could be due to the different plant tissues’ characteristics; in the laboratory, we inoculated detached leaf tissues that were kept in Petri dishes for 21 days, while in the field the oospores initially differentiate on living leaves that receive nutrients from the plant and probably, in these conditions, the pathogen has more energy for the sexual reproduction process. The very low number of oospores retrieved from the laboratory samples did not allow us to assess their germinability and infectivity. Indeed, the oospores normally germinate at a relatively low percentage (0.1–22%) [36,37,38], and probably a higher number of oospores could have helped us in assessing this very important parameter. Despite this, the results obtained in this study highlight that *P. viticola* had potential penalties in the oospore production on resistant cultivars in one crossing (CASB11xCAST7) out of three. This is partly in line with the results reported in a previous study on samples collected in the field, where the oospore production was four times higher on *V. vinifera* cultivars than on plants harboring *Rpv1* and *Rpv3* loci [39]. It must be pointed out that, in our case, we used single isolates weekly propagated on detached leaves to produce oospores and the absence of significant differences in the density on the different cultivars could be originated either from the low amount of oospores produced or the different characteristics of the isolates, which should be better investigated. The viability of the oospores differentiated in vineyards and overwintered in the same controlled conditions adopted in this study, ranged from 10–40% [36]. The very high values (67–93%) obtained in the viability tests in laboratory conditions showed that most of the oospores successfully completed their maturation period, reaching a stable structure, while few oospores degenerated. This could be due to the fact that the oospores were kept in controlled environmental conditions, while in the field the oospores undergo variable environmental conditions that can negatively affect their maturation process and viability at the initial stages. Since the oospores formed by the different mating pairs possessed high and analogous viability on all the cultivars, our results confirm that resistant cultivars seem to act on the differentiation more than on the viability of the oospores, as already observed [39]. In the future, it would be useful to enlarge the collection and characterization of *P. viticola* oospores, differentiated both in the field and laboratory, for their amount, viability, germinability, and infectivity. The study of field-differentiated oospores has the advantage of investigating the response of the different cultivars to natural conditions, while laboratory crosses allow us to precisely evaluate the effect of the cultivars on the individual mating pairs. An improvement in the protocol for the experimental production of oospores in the laboratory (e.g., by inoculating *P. viticola* strains on plants or increasing the number of inoculated leaves) is, however, necessary to achieve enough material for all such experimental activities.

## 4. Materials and Methods

### 4.1. Plant Material

Potted plants of Cabernet sauvignon (susceptible *V. vinifera* variety), Pinot noir (PN, susceptible *V. vinifera* variety), Mgaloblishvili (MG, resistant *V. vinifera* variety), and Bianca (B, resistant interspecific hybrid) were grown in the greenhouse (24 °C, 16 h photoperiod, 70% relative humidity) of the University of Milan at the Department of Agricultural and Environmental Science (DISAA). Cabernet sauvignon was used for the isolation and propagation of *P. viticola* isolates, while Pinot noir, Mgaloblishvili and Bianca were used for evaluating *P. viticola* pathogenicity and sexual reproduction.

### 4.2. P. viticola Isolates

**Figure 5:**
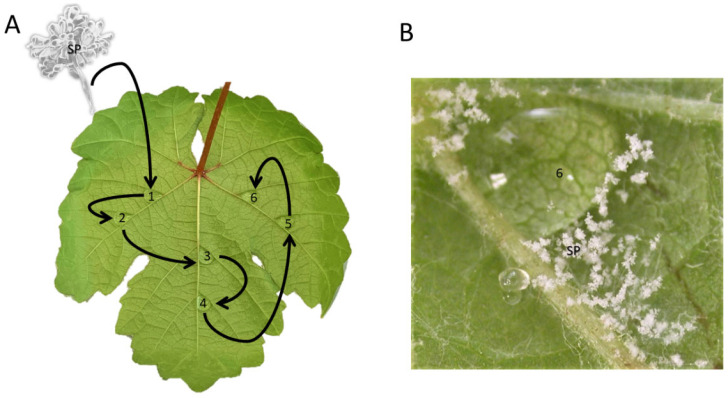
*P. viticola* isolation procedure. (**A**) An individual sporangiophore (SP) was inoculated in a 25 µL water droplet (droplet n.1) and serially diluted five times (until droplet n.6). (**B**) Seven days after incubation of the inoculated leaf at 22 °C (16 h of light/day), sporangiophores (SP, in white) were taken from the last droplet (n.6) and inoculated on fresh leaves to propagate the isolate.

Leaves showing downy mildew symptoms and sporulation were sampled from 14 vineyards located in five North Italian regions in spring–summer 2019 (Table 6). The samples (50 leaves/vineyard) were placed in plastic bags and transported to the laboratory in refrigerated conditions. To promote fresh sporulation, leaves were washed under running tap water and kept in a humid chamber at 22 °C overnight [4]. Each leaf was examined at the stereomicroscope Leica Wild M10 (40–60×) to individuate and detach a single sporangiophore with sterile forceps [40]. A grapevine leaf (cv Cabernet sauvignon) was placed, with the abaxial surface upwards, inside a Petri dish containing moistened filter paper. Six drops of 25 µL of sterile distilled water were placed on the leaves. The isolated sporangiophore was inoculated on the first droplet and serially diluted 5 times by transferring 5 µL of sporangia suspension to the subsequent droplet (Figure 5A). Serial dilution was performed to obtain isolates from single or very few sporangia. The inoculated leaves were incubated in a growth chamber at 22 °C (16 h of light/day) for seven days. The sporangia formed in the least concentrated droplet (Figure 5B) were then collected in 200 µL of sterile distilled water and inoculated onto a fresh leaf as described in paragraph 2.3 to propagate the isolate. A total of 102 strains of *P. viticola* were isolated but only 72 survived and were weekly propagated on healthy leaves of cv Cabernet sauvignon.

### 4.3. Experimental Inoculation and Pathogenicity Evaluation

The third to the fifth leaves starting from the apex of the shoots of the plants were sampled for the experimental inoculations. A single leaf was collected from three different plants and three leaf discs (1.5 cm diameter) per leaf were excised with a cork borer and placed, with the abaxial surface upwards, in Petri dishes containing moistened filter paper. For isolate propagation, three 10 µL droplets of sporangia suspension (5 × 10^4^ sporangia/mL) were inoculated onto the leaf discs of Cabernet sauvignon. For pathogenicity assays, a single 10 µL droplet of sporangia suspension (5 × 10^4^ sporangia/mL) was inoculated in the center of each PN, MG, and B leaf disc. The inoculated leaves were incubated in a growth chamber at 22 °C (16 h of light/day) and scored for the disease intensity at 5, 7, 9, and 13 days post inoculation (dpi). Infection frequency (IF), a measure of disease incidence, was calculated as the proportion of sporulating sites over the total inoculated droplets [27]. Disease severity was estimated by image analysis of the leaf area covered by sporulation and expressed as a percentage of the sporulating area (PSA) [41]. The reduction in PSA on B and MG compared to PN (RP, expressed as a percentage) was calculated at 13 dpi as $RP=100-\left(\frac{PSA_{x}}{PSA_{PN}}\times 100\right)$ where *x* indicates either B or MG. RP values were used to identify strains that are able to overcome B and MG resistance (adapted strains) following this scheme: the non-adapted strains (i.e., those showing no or limited growth on resistant cultivars) should possess the highest RP values, while the adapted strains should possess the least RP values. The RP values of adapted/non-adapted strains were selected based on the data distribution. The latency period (LP) duration was estimated as the number of days between inoculation and first sporulation observation, expressed as degree-days (DD) by multiplying the day by the incubation temperature (22 °C) [42]. The isolates were classified into four pathogenicity classes (PC) based on the presence of sporulation on the leaf tissues at 13 dpi. PC1 grouped strains with sporulation only on PN; PC2 sporulation on PN and B and absence of sporulation on MG; PC3 sporulation on PN and MG and absence of sporulation on B; and PC4 presence of sporulation on PN, B and MG.

### 4.4. Oospore Production and Viability

*P. viticola* is a biotrophic pathogen that overwinters as sexual spores, the oospores. Since *P. viticola* is heterothallic, two compatible mating types are needed to produce the oospores [43,44,45]. The mating compatibility was assessed by co-inoculating two strains at a time on leaf discs of cv Cabernet sauvignon as described for the experimental inoculations [45]. The mating was considered successful when oospores could be observed at the optical microscope (Zeiss Primo Vert) at 14–21 dpi [43]. Mating-type tests were carried out in triplicate. Successful mating pairs individuated within PC4 (CAST1, CAST7, and CASB11) were employed for oospore production. Sporangia suspensions from two strains were mixed in a 1:1 ratio and six 25 µL droplets from the mixed suspension were inoculated on ten detached leaves of PN, B, and MG placed with the abaxial surface upwards. For each cross and each grapevine variety, 60 leaf discs (11 mm diameter) were cut from the leaves with a cork borer at 21 dpi, divided into three different nylon bags (100 µm pores) and overwintered for 4 months at 5 °C, 30% RH [36]. The oospores were isolated from the leaf tissues contained in each nylon bag by using a glass Potter, suspended in water, and counted in Kova chambers at the optical microscope (Zeiss Primo Vert). Three replicates of 10 µL water suspension were considered per sample. The total number of oospores was calculated by multiplying the oospore concentration in 10 µL by the total volume of oospore suspension. The oospore density (OD), expressed as oospores/cm^2^ of leaf tissue, was calculated by dividing the total number of oospores by the leaf disc area [35].

Oospore viability (OV) was assessed by using trypan blue staining [36]. Briefly, oospores were incubated for 5 min at 90 °C in 1 mg/mL trypan blue solution (1 mg trypan blue dissolved in 1 mL of lactoglycerol water 1:1 *v*:*v*). The oospores were de-stained twice for 2 h at room temperature in a 2.5 g/mL chloralium hydrate water solution, and resuspended in water to count the number of blue-colored (dead) and unstained (living) oospores on three replicates of 30 oospores at the optical microscope (Zeiss Primo Vert). Results were reported as the percentage of viable oospores (OV).

### 4.5. Data Analysis

The PSA response variation along the experimental timeframe of investigation (*x* = dpi) was evaluated through a three-parameter log-logistic regression model by using the following formula:

(1) $$f\left(x\right)=\frac{d}{1+\exp\left(b\left(\log\left(x\right)-\log(e\right)\right)}$$

where *b* represents the slope of the curve (it describes how rapidly the curve makes its transition between the two asymptotes or it describes how rapidly the response variable reaches its maximum level); *d* is the value of the horizontal upper asymptote when *x* -> +∞ (it describes the maximum level reached by the response variable); *e* represents the x value at the curve inflection point. The lower asymptote (*x* -> -∞) was fixed to 0. PSA data were transformed as *y* = log(PSA + 1) and the logistic model was fitted using the LL.3() function implemented in the drc package [46]. Simultaneous inference of estimated parameters was evaluated according to t-statistic *p*-values using the summary.drc() and a lack-of-fit test was applied to assess the model fit [47]. The log-logistic model was fitted by grouping the independent variable on grapevine cultivar. Estimated parameters from the different curves obtained with the model were then compared by means of a z-test using the function drc::compParm(). Response time, i.e., time to reach 50% of the total PSA (t_50_), was calculated using ED.drc(), and comparisons among t_50_ values were estimated through EDcomp() function in drc. t_50_ (time to reach 50% of disease) is one of the most important parameters used in plant disease epidemiology to describe disease progress [48].

A Spearman correlation test was performed on PC, province, and vineyard of origin of the strains. The existence of significant differences among the rank-transformed LP, IF, and PSA values recorded by the isolates and PCs on the three cultivars at each dpi was assessed by Kruskal–Wallis test and pairwise comparison with SPSS v. 26 (IBM Analytics, Milano, Italy).

ANOVA was performed on oospore density (OD), divided according to mating pairs and grapevine cultivar (SPSS v.26). Fisher’s exact test was used to determine if there was a significant association between oospore viability, expressed as the count of stained and unstained cells, and (i) cultivar or (ii) mating pair. To perform the analyses, fisher.test() function in R (v3.6) was employed.

## 5. Conclusions

In conclusion, the data provided in the present study indicate that infection frequency and severity, the parameters that are most strongly indicative of a plant’s response to the pathogen [27], were significantly reduced by the two resistant cultivars, in a measure that is compatible with the different resistance levels normally imposed by the two varieties (disease severity <25% in MG and <5% on B) [14,17,19]. Very few strains were able to reach significant growth on the two resistant cultivars, and only two strains showed high infectivity on MG in the presence of high virulence on PN. This indicates that, in general, good protection can be achieved by these varieties. The signatures of fitness penalties (reduced virulence, infection frequency, and oospore production) indicate that the strains able to grow on resistant cultivars could be only slightly less competitive than non-adapted *P. viticola* strains and may potentially diffuse in vineyards. The absence of evidence for geographical distribution within PCs indicates that the strains able to infect resistant cultivars are widespread over the monitored territory, where several cultivars with *Rpv3* QTL are cultivated for wine production. Therefore, proper management of resistant grapevines must be carried out in vineyards, by adopting all the measures that prevent the selection and diffusion of adapted strains and protect these important resistant genes from erosion [23,49].

## Figures and Tables

**Table 1:** Percentage of infection frequency (IF) and percentage of sporulating area (PSA) ± standard deviation (SD) recorded by *P. viticola* strains grouped or not into pathogenicity classes (PC) on Pinot noir, Mgaloblishvili, and Bianca. Different letters within columns indicate the existence of significant differences among the IF and PSA values of the different PC classes (*p* < 0.05).

Grapevine cv	Variable		dpi			
		PC	5	7	9	13
Pinot noir	IF (%) ± SD	1	30 ± 30 a	89 ± 30 b	98 ± 8 c	100 ± 0 c
		2	63 ± 38 a	92 ± 15 ab	92 ± 15 bc	92 ± 15 abc
		3	33 ± 32 a	56 ± 33 a	53 ± 32 a	67 ± 25 a
		4	44 ± 34 a	69 ± 29 ab	70 ± 26 ab	80 ± 26 ab
		all isolates	41 ± 37	74 ± 31	77 ± 27	84 ± 24
	PSA (%) ± SD	1	0.5 ± 0.5 a	4 ± 4 a	13 ± 13a	30 ± 30 a
		2	2 ± 2 a	8 ± 5 a	26 ± 14 a	48 ± 31 a
		3	1.4 ± 1.4 a	6 ± 6 a	16 ± 10 a	47 ± 30 a
		4	2 ± 2 a	7 ± 7 a	19 ± 15 a	38 ± 20 a
		all isolates	1 ± 1	6 ± 6	18 ± 14	39 ± 27
Mgaloblishvili	IF (%) ± SD	1	-	-	-	-
		2	-	-	-	-
		3	25 ± 25 a	50 ± 27 a	53 ± 26 a	67 ± 32 a
		4	34 ± 32 a	55 ± 29 a	56 ± 27 a	76 ± 25 a
		all isolates	20 ± 20	33 ± 33	34 ± 34	46 ± 42
	PSA (%) ± SD	1	-	-	-	-
		2	-	-	-	-
		3	0.4 ± 0.4 a	3 ± 3 a	5 ± 5 a	15 ± 11 a
		4	0.7 ± 0.7 a	3 ± 3 a	7 ± 7 a	23 ± 16 a
		all isolates	0.4 ± 0.4	2 ± 2	4 ± 4	13 ± 13
Bianca	IF (%) ± SD	1	-	-	-	-
		2	29 ± 27 a	42 ± 30 a	50 ± 31 a	54 ± 25 a
		3	-	-	-	-
		4	24 ± 22 a	43 ± 26 a	48 ± 26 a	54 ± 26 a
		all isolates	14 ± 14	25 ± 25	28 ± 28	31 ± 31
	PSA (%) ± SD	1	-	-	-	-
		2	0.5 ± 0.5 a	0.5 ± 0.5 a	1 ± 1a	1.7 ± 1.7 a
		3	-	-	-	-
		4	0.1 ± 0.1 a	0.4 ± 0.4 a	0.8 ± 0.8 a	1.1 ± 1.1 a
		all isolates	0.1 ± 0.1	0.3 ± 0.3	0.5 ± 0.5	0.7 ± 0.7

**Table 2:** Estimated parameters obtained for each curve (grapevine cultivar) with the log-logistic model on transformed PSA values. Different letters within columns indicate the existence of significant differences among log(x + 1) transformed *b* (slope) and *d* (upper asymptote) values and *e* (t_50_) values (expressed as dpi) (*p* < 0.01). Values in brackets represents back-transformed (y = e^x^ + 1) values of *d* to the original scale (PSA %).

Grapevine Cultivar	Model Parameters		
	*b*	*D*	*e* (dpi)
Pinot noir	−4.53 a	3.61 (38) b	7.39 a
Mgaloblishvili	−3.53 ab	2.13 (9.4) a	8.95 a
Bianca	−1.50 b	3.26 *	51.42 *

* *t*-statistics *p* > 0.05.

**Table 3:** Number and percentage (in brackets) of strains grouped in different PSA ranges on Pinot noir, Bianca and Mgaloblishvili.

PSA Range	Grapevine Variety		
	Pinot Noir	Bianca	Mgaloblishvili
0	0	32 (44%)	27 (38%)
0.1–5%	7 (10%)	38 (53%)	7 (10%)
5.1–10%	4 (5%)	2 (3%)	7 (10%)
10.1–25%	16 (22%)	0	13 (18%)
25.1–50%	23 (32%)	0	16 (22%)
50.1–75%	14 (20%)	0	2 (2%)
75.1–100%	8 (11%)	0	0

**Table 4:** Pathogenicity classes * assigned to the isolates.

Pathogenicity Class	Isolates	Number and Percentage (in Brackets) of Isolates
1	BET4-BET6-CASB1-CASB16-CASB5-CASB9-CAST10-REF.F12-REF13-REF1A-REF1E-REF9-SAC9-ZEN9A-ZIN2-ZOX11.2.2-ZOX16.1.1-ZOX16.29-ZOX20.2.9	19 (26%)
2	CASB12-CASB28-CASB3-SAC2-SAC6-ZIN4-ZOX11.1.2.4-ZOX6.19	8 (11%)
3	BEB6-BET3-CASB30-CASB31-CASB51-CAST12-CAST8-REF12-REF8-ZIN7-ZOX14.2.3-ZOX16.2.1	12 (17%)
4	BEB3-BET17-CASB11-CASB13-CASB14-CASB15-CASB18-CASB23-CASB36-CASB4-CASB6-CAST1-CAST3-CAST6-CAST7-CAST9-REF11-REF14-REF3A-REF3C-SAC3-SAC4-VILLO6-ZEN3-ZIN1-ZIN6-ZIN8-ZOX16.1.8-ZOX16.2.6-ZOX20.11-ZOX20.2.2-ZOX6.1.1.1-ZOX6.1.8	33 (46%)

* Class 1: isolates infecting only PN. Class 2: isolates infecting PN and B; Class 3: isolates infecting PN and MG. Class 4: isolates infecting all the cultivars.

**Table 5:** Density (OD) and viability (OV) of *P. viticola* oospores differentiated by the three different mating pairs (CASB11xCAST1, CASB11xCAST7, and CAST1xCAST7) on the susceptible (Pinot noir) and resistant (Bianca and Mgaloblishvili) grapevine varieties. Different letters indicate the existence of significant differences among cultivars (*p* < 0.05).

Mating Pairs	OD (oospores/cm^2^)			OV (%)		
	Pinot Noir	Bianca	Mgaloblishvili	Pinot Noir	Bianca	Mgaloblishvili
CAST1xCAST7	7.1 ± 2	6.3 ± 2	4.9 ± 1,4	67 ± 15	80 ± 26	70 ± 10
CASB11xCAST1	6 ± 1.7	5.9 ± 1.6	5.2 ± 0.9	77 ± 15	83 ± 15	77 ± 15
CASB11xCAST7	8.2 ± 1.9 b	4.6 ± 0.4 a	5.6 ± 0.6 a	87 ± 15	93 ± 12	87 ± 15

**Table 6:** Location of the vineyards and list of *P. viticola* isolates.

Vineyard N.	Vineyard Location	Region	Name of the Isolates	Number of Isolates per Vineyard
1	Santa Maria della Versa (PV)	Lombardy (NW)	BEB3, BEB6	2
2			BET3-BET4-BET6-BET17	4
3	Casarsa della Delizia (PN)	Friuli-Venezia Giulia (NE)	CASB1-CASB3-CASB4-CASB5-CASB6-CASB9-CASB11-CASB12-CASB13-CASB14-CASB15-CASB16-CASB18-CASB23-CASB28-CASB30-CASB31-CASB36-CASB51	19
4			CAST1-CAST3-CAST6-CAST7-CAST8-CAST9-CAST10-CAST12	8
5	Refrontolo (TV)	Veneto (NE)	REF1A-REF1E-REF3A-REF3C-REF8-REF9-REF11-REF.F12-REF13-REF14-REF12	11
6	Fognano di Brisighella (RA)	Emilia Romagna (NE)	SAC2-SAC3-SAC4-SAC6-SAC9	5
7	Villorba (TV)	Veneto (NE)	VILLO6	1
8	Zenson di Piave (TV)	Veneto (NE)	ZEN9A, ZEN3	2
9	Casarsa della Delizia (PN)	Friuli-Venezia Giulia (NE)	ZIN1-ZIN2-ZIN4-ZIN6-ZIN7-ZIN8	6
10	Valeggio sul Mincio (VR)	Veneto (NE)	ZOX6.1.8-ZOX6.1.1.1-ZOX6.19	3
11	Roverè della Luna (TN)	Trentino-Alto Adige (NE)	ZOX11.1.2.4-ZOX11.2.2	2
12	Valdobbiadene, f. San Giovanni (TV)	Veneto (NE)	ZOX14.2.3	1
13	Vidor di Valdobbiadene (TV)	Veneto (NE)	ZOX16.2.6-ZOX16.2.1-ZOX16.1.1-ZOX16.1.8-ZOX16.29	5
14	Valdobbiadene (TV)	Veneto (NE)	ZOX20.2.2-ZOX20.2.9-ZOX20.11	3

## Data Availability

The data presented in this study are available on request from the corresponding author.

## Supplementary Materials

The following supporting information can be downloaded at: https://www.mdpi.com/article/10.3390/plants11192619/s1, Table S1: List of *P. viticola* strains with their PSA (percentage of sporulating area) on Pinot noir, RP (reduction of PSA on Bianca and Mgaloblishvili compared to PN), expressed as percentages, and classification in adapted (A) or non-adapted (NA) strains according to RP < 85% for Bianca and RP < 25% for Mgaloblishvili.
