# Supplementary material for: Pathogen Adaptation to American (Rpv3-1) and Eurasian (Rpv29) Grapevine Loci Conferring Resistance to Downy Mildew
Source: Plants (Basel). 2022 Oct 5;11(19):2619. doi: 10.3390/plants11192619 (PMC9571346; doi:10.3390/plants11192619)
Supplement: Supplementary file 1 [file plants-11-02619-s001.zip › plants-1884617-supplementary.pdf]

Supplementary Table S1. List of *P. viticola* strains with their PSA (percentage of sporulating area) on Pinot noir, RP (reduction of PSA on Bianca and Mgaloblishvili compared to PN), expressed as percentages, and classification in adapted (A) or non-adapted (NA) strains according to RP<85% for Bianca and RP<25% for Mgaloblishvili.

Strain	PSA (%)	RP (%)		Adaptation	
	Pinot noir	Bianca	Mgaloblishvili	Bianca	Mgaloblishvili
CAST7	7.2	74.6	0	A	A
CASB11	15.2	99.2	0	NA	A
CAST3	22.5	99.1	0	NA	A
CASB6	22.6	95	0	NA	A
REF14	22.7	92.6	0	NA	A
SAC4	25.4	96.4	0	NA	A
ZOX20.11	26.4	99.7	0	NA	A
ZIN8	26.7	93.3	0	NA	A
ZOX16.2.6	74.7	99.9	11.1	NA	A
REF3A	62.4	98.8	18.2	NA	A
BEB3	56.6	83.5	47.6	A	NA
SAC3	19.6	77.3	51.3	A	NA
CAST9	3.5	76.6	89.3	A	NA
ZOX11.1.2.4	0.8	79	100	A	NA
REF11	41.5	99.4	32.8	NA	NA
REF12	42	100	34	NA	NA
ZEN3	78.2	99.8	36	NA	NA
CASB30	54.5	100	36.5	NA	NA
CASB23	61.2	96.5	36.9	NA	NA
REF8	40	100	45.7	NA	NA
BET17	42	95.8	46.6	NA	NA
VILLO6	69.5	98.2	47.1	NA	NA
ZIN6	37.1	93.6	48.9	NA	NA
ZOX6.1.1.1	35	95.4	49.3	NA	NA
CASB18	24.1	98.1	52.3	NA	NA
ZOX16.1.8	62.1	99	52.6	NA	NA
CASB13	58	99.6	53.5	NA	NA
ZOX16.2.1	12.3	100	58.8	NA	NA
CASB31	65.8	100	61.9	NA	NA
ZOX14.2.3	28.3	100	63.1	NA	NA
ZIN7	38.8	100	66.3	NA	NA
CAST8	63.3	100	66.7	NA	NA
CASB14	31.8	98.9	71.4	NA	NA
ZOX20.2.2	44.4	98.2	74	NA	NA
REF3C	47.4	99.9	78.2	NA	NA
CASB15	26	98.1	78.7	NA	NA
ZOX6.1.8	3.4	100	79.9	NA	NA

ZIN1	53.9	99.7	80	NA	NA
BET3	11.8	100	84.5	NA	NA
CASB4	18.7	99.7	85.7	NA	NA
CAST1	49.9	98.6	88.3	NA	NA
CASB51	96.1	100	89.8	NA	NA
BEB6	8.8	100	92.3	NA	NA
CAST6	35.5	99.5	92.9	NA	NA
CAST12	97.3	100	94.3	NA	NA
CASB36	53.9	98.9	96.8	NA	NA
SAC9	0.5	100	100	NA	NA
REF.F12	2.1	100	100	NA	NA
ZOX11.2.2	2.3	100	100	NA	NA
CASB1	2.8	100	100	NA	NA
CASB9	8.2	100	100	NA	NA
REF9	9.5	100	100	NA	NA
ZEN9A	10.7	100	100	NA	NA
REF1E	16.6	100	100	NA	NA
REF13	17.6	100	100	NA	NA
CAST10	18.6	100	100	NA	NA
CASB12	20.6	94.7	100	NA	NA
REF1A	20.9	100	100	NA	NA
ZOX16.1.1	24.3	100	100	NA	NA
BET6	25.1	100	100	NA	NA
SAC6	34.9	99.2	100	NA	NA
CASB16	40.9	100	100	NA	NA
CASB3	44.5	99.3	100	NA	NA
BET4	46.6	100	100	NA	NA
ZOX6.19	49.6	94.3	100	NA	NA
ZOX16.29	51.5	100	100	NA	NA
SAC2	53.6	99.2	100	NA	NA
CASB28	84.1	98.2	100	NA	NA
ZIN2	86.1	100	100	NA	NA
CASB5	92.2	100	100	NA	NA
ZIN4	95.9	93	100	NA	NA
ZOX20.2.9	96.8	100	100	NA	NA
